# Multiple myeloma with G‐CSF production mimicking chronic neutrophilic leukemia

**DOI:** 10.1002/jha2.171

**Published:** 2021-03-09

**Authors:** Hiroshi Ureshino, Koichi Ohshima, Masaharu Miyahara

**Affiliations:** ^1^ Division of Hematology, Respiratory Medicine and Oncology, Department of Internal Medicine Faculty of Medicine Saga University Saga Japan; ^2^ Department of Internal Medicine Karatsu Red Cross Hospital Karatsu Japan; ^3^ Department of Pathology Kurume University Kurume Japan

A 68‐year‐old man with a history of multiple myeloma (IgG subtype, stage II by the international staging system) following autologous stem cell transplantation received a 9‐year maintenance therapy with lenalidomide (maintenance of complete remission). Upon follow‐up, severe leukocytosis was documented in June, 2020 (white cell count, 55.6 × 10^9^/L without blasts). A bone marrow aspirate revealed hypercellularity with primarily mature neutrophils (Figure 1 **A and B**) and a small aggregation of plasma cells (Figure 1 **C**). No *BCR‐ABL1* transcript was detected. An initial diagnosis of chronic neutrophilic leukemia was made. Hydroxyurea was initiated, and leukocytosis gradually diminished. Subsequently, the patient complained of a right‐sided chest lump in June, 2020. Biopsy of the chest wall mass was performed, revealing a plasmacytoma with positive CD38 (Figure 1 **D**) and granulocyte colony‐stimulating factor (G‐CSF) staining (Figure 1 **E**). Serum G‐CSF levels were also increased (4950 pg/ml; normal range, <39 pg/ml); thus, a diagnosis of relapsed multiple myeloma with G‐CSF production was established. Pomalidomide and dexamethasone therapy was initiated, and serum G‐CSF levels decreased (788 pg/ml), and leukocyte counts were almost normalized 1 month after follow‐up.

**FIGURE 1:**
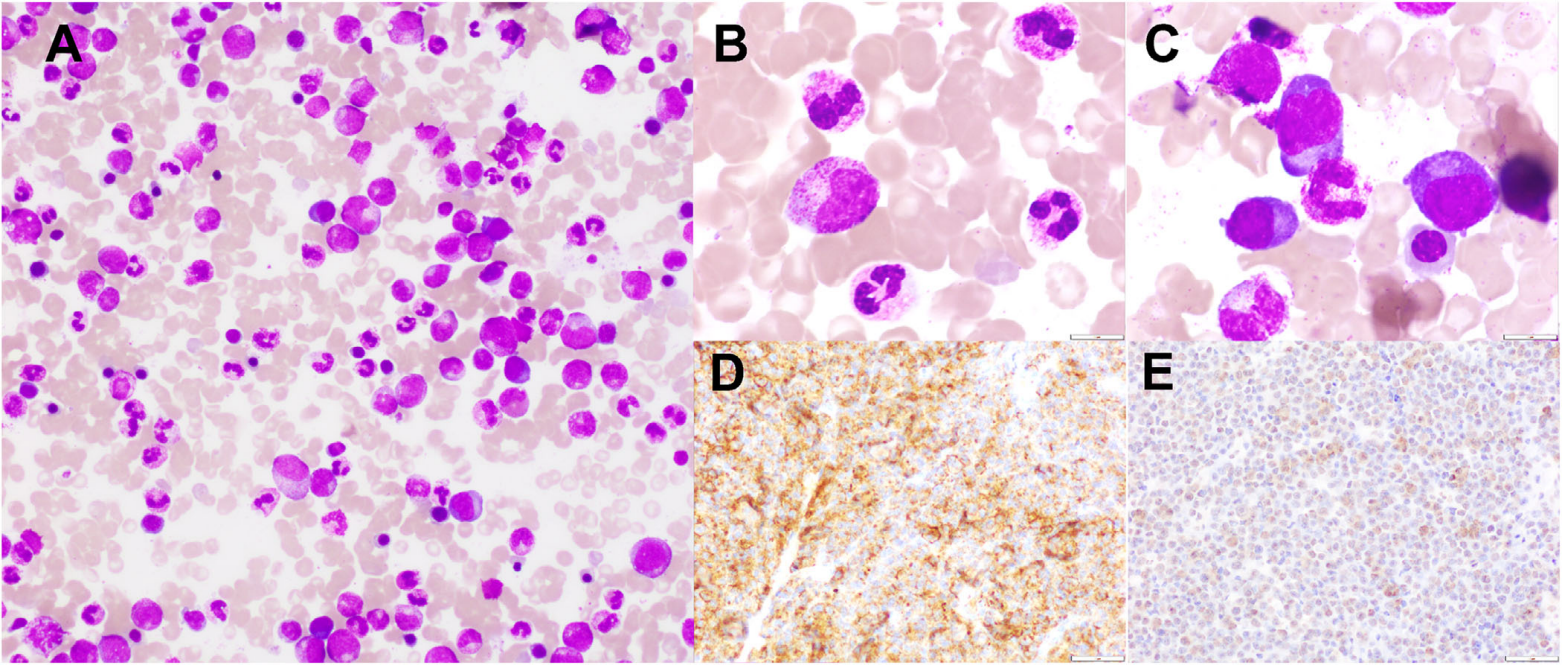
Findings of bone marrow aspirate. Mature neutrophils (A and B) and a small aggregation of plasma cells (C) were observed. May‐Giemsa stain: (A) magnification 100×; (B and C) 200×. Pathological findings of biopsied chest wall mass. Plasmacytoma with CD38 (D) and granulocyte colony‐stimulating factor (G‐CSF) staining (E) positive were observed; CD38 and G‐CSF staining; magnification 100×

Severe leukocytosis (>50 × 10^9^/L) generally can be caused by acute leukemia or myeloproliferative neoplasms. Other causes are reported as leukemoid reactions consisting severe infections, adverse drug reactions or G‐CSF producing tumors. G‐CSF elicits proliferation and activation of hematopoietic stem cell of the granulocyte lineage, leading to occurrence of severe leukocytosis. G‐CSF producing tumor is rare, and generally reported in patients with solid tumors. Although G‐CSF producing multiple myeloma is extremely rare, G‐CSF produced by multiple myeloma cells in the case can be the cause of severe leukocytosis, mimicking chronic neutrophilic leukemia. Physicians should pay attention for the rare disease when the severe leukocytosis appears.

## AUTHOR CONTRIBUTIONS

Hiroshi Ureshino and Masaharu Miyahara provided care for the patient or reviewed the case. Koichi Ohshima provided pathological diagnosis. Hiroshi Ureshino wrote the initial draft of this manuscript. All authors approved the final version. Written consent for publication was obtained from the patient.

## CONFLICT OF INTEREST

The authors declare no conflicts of interest.

